# Contents, Followers, and Retweets of the Centers for Disease Control and Prevention’s Office of Advanced Molecular Detection (@CDC_AMD) Twitter Profile: Cross-Sectional Study

**DOI:** 10.2196/publichealth.8737

**Published:** 2018-04-02

**Authors:** Isaac Chun-Hai Fung, Ashley M Jackson, Lindsay A Mullican, Elizabeth B Blankenship, Mary Elizabeth Goff, Amy J Guinn, Nitin Saroha, Zion Tsz Ho Tse

**Affiliations:** ^1^ Department of Epidemiology and Environmental Health Sciences Jiann-Ping Hsu College of Public Health Georgia Southern University Statesboro, GA United States; ^2^ Office of Advanced Molecular Detection National Center for Emerging and Zoonotic Infectious Diseases Centers for Disease Control and Prevention Atlanta, GA United States; ^3^ Department of Computer Science The University of Georgia Athens, GA United States; ^4^ School of Electrical and Computer Engineering College of Engineering The University of Georgia Athens, GA United States

**Keywords:** communications media, health communication, social media

## Abstract

**Background:**

The Office of Advanced Molecular Detection (OAMD), Centers for Disease Control and Prevention (CDC), manages a Twitter profile (@CDC_AMD). To our knowledge, no prior study has analyzed a CDC Twitter handle’s entire contents and all followers.

**Objective:**

This study aimed to describe the contents and followers of the Twitter profile @CDC_AMD and to assess if attaching photos or videos to tweets posted by @CDC_AMD would increase retweet frequency.

**Methods:**

Data of @CDC_AMD were retrieved on November 21, 2016. All followers (N=809) were manually categorized. All tweets (N=768) were manually coded for contents and whether photos or videos were attached. Retweet count for each tweet was recorded. Negative binomial regression models were applied to both the original and the retweet corpora.

**Results:**

Among the 809 followers, 26.0% (210/809) were individual health professionals, 11.6% (94/809) nongovernmental organizations, 3.3% (27/809) government agencies’ accounts, 3.3% (27/809) accounts of media organizations and journalists, and 0.9% (7/809) academic journals, with 54.9% (444/809) categorized as miscellaneous. A total of 46.9% (360/768) of @CDC_AMD’s tweets referred to the Office’s website and their current research; 17.6% (135/768) referred to their scientists’ publications. Moreover, 80% (69/86) of tweets retweeted by @CDC_AMD fell into the miscellaneous category. In addition, 43.4% (333/768) of the tweets contained photos or videos, whereas the remaining 56.6% (435/768) did not. Attaching photos or videos to original @CDC_AMD tweets increases the number of retweets by 37% (probability ratio=1.37, 95% CI 1.13-1.67, *P*=.002). Content topics did not explain or modify this association.

**Conclusions:**

This study confirms CDC health communicators’ experience that original tweets created by @CDC_AMD Twitter profile sharing images or videos (or their links) received more retweets. The current policy of attaching images to tweets should be encouraged.

## Introduction

Twitter (San Francisco, CA) has been used by public health practitioners for purposes ranging from public health surveillance [1] to health communication [2,3] to natural disaster preparedness [4]. A recent systematic review identified taxonomy of Twitter as a tool for health research, including content analysis, surveillance, engagement, recruitment, intervention, and network analysis [5]. Prior research found Twitter users engaged in communication pertinent to infectious diseases, such as Ebola [6,7], measles [8], Middle East Respiratory Syndrome [9], and Zika [10,11]. The Centers for Disease Control and Prevention (CDC) encourages the strategic use of Twitter to disseminate CDC health information and engage with individuals and partners [12]. CDC uses Twitter as part of their overall health communication strategies [13,14], uses Twitter chats to engage Twitter users on specific health topics [15-17], and publicizes public health events and publications via Twitter [18].

Advanced molecular detection (AMD) harnesses the power of next-generation genomic sequencing, high-performance computing, and epidemiology to study pathogens. CDC uses AMD technologies to identify emerging pathogens, improve vaccines, make food safer, develop faster tests, and connect information from public health investigations with genomic data from pathogens to understand how infections spread [19].

The Office of Advanced Molecular Detection (OAMD) in the National Center for Emerging and Zoonotic Infectious Diseases (NCEZID), CDC, promotes open sharing of scientific data through publicly accessible platforms and added Twitter (@CDC_AMD) as a communication channel in May 2015. OAMD uses Twitter to promote free exchange of information and interactivity between CDC infectious disease programs and partners in federal and state agencies, academia, and professional organizations, as well as the general public. OAMD noted the value of Twitter in March 2016, when a tweet regarding CDC’s submission of genomic sequence data to a publicly accessible database led to a global discussion on *Elizabethkingia anophelis* and a subsequent international partnership to investigate this rare bacterium [20].

In this case study, we analyzed the @CDC_AMD Twitter handle to divulge information regarding followers and popular tweet content. Here, we address 3 research questions (RQ):

RQ1: Who are the followers of @CDC_AMD?

RQ2: What content has been tweeted the most?

RQ3: Does attaching a photo or video (or a link to a photo or video) increase the probability ratio of a tweet being retweeted?

This case study enables us to understand how a CDC Twitter account communicates scientific information to its followers, and provides health communicators with information for future enhancement of their Twitter communication.

## Methods

### Data

Data were retrieved via Twitter Search Application Programming Interface and downloaded to a server at Athens, Georgia, USA. Tweets tweeted by OAMD from the first tweet on May 5, 2015, 3:04 pm (Universal Coordinated Time, UTC) to the 768th tweet on November 16, 2016, 3:22 pm UTC were retrieved. Data on favorites, followers, followings, and tweets, including retweet count, were downloaded by November 21, 2016. A protocol for analysis was created and approved for use by OAMD.

CDC encourages the strategic use of Twitter to disseminate CDC health information and engage with individuals and partners. OAMD’s Twitter guidelines, based on CDC’s Twitter Guidelines and Best Practices [12], give careful consideration to the nature of @CDC_AMD Twitter messages and activities. All tweets posted by @CDC_AMD are cleared internally by OAMD scientific and communication staff or cross-cleared with scientific and communication staff in other CDC programs that are conducting AMD-related activities.

### Manual Coding

First, we manually categorized @CDC_AMD’s followers into 6 categories specified by OAMD:

1. Individual scientists, physicians, and other public health professionals
2. Governmental organizations, such as other branches in CDC, and other federal agencies
3. Nongovernmental organizations, such as a scientific society
4. Mass media organizations and their representatives, such as Cable News Network, and individual journalists
5. Academic journals, such as *Journal of the American Medical Association* and *Science*
6. Miscellaneous: anyone who did not belong to the aforementioned categories

Next, we manually categorized @CDC_AMD’s tweets into 6 categories specified by OAMD:

1. Tweets that refer to CDC’s AMD website [21]
2. Tweets that refer to publications of CDC’s AMD scientists (usually their abstract on PubMed)
3. Training: announcement of webinars, every quarter, in collaboration with Association of Public Health Laboratories (APHL)
4. Training: announcement of CDC Bioinformatics fellowship program, in collaboration with APHL
5. CDC AMD scientists’ activities, such as their visit to a state laboratory
6. Miscellaneous: anything that does not belong to the aforementioned categories

Then, we manually determined whether photos or videos, or their links, were attached to the tweets. We also manually coded whether a tweet was an original tweet posted by @CDC_AMD or it was a retweet by @CDC_AMD of a tweet originally posted by other Twitter users.

A total of 10% random samples of original tweets posted by @CDC_AMD (68/682), @CDC_AMD’s retweets of other Twitter users’ tweets (9/86), and followers of @CDC_AMD (81/809) were double-coded by 2 independent coders, and the inter-rater reliability is substantial (κ=.749, .757, and .839, respectively) [22].

### Statistical Analysis

Statistical analysis was conducted in R 3.3.1 (R Core Team, R Foundation for Statistical Computing, Vienna, Austria) via RStudio 0.99.903 (RStudio, Inc., Boston, MA). For Poisson regression models, we used glm() in stats package. For negative binomial regression models, we used glm.nb() in MASS package. For hurdle models, we used hurdle() in pscl package [23].

We stratified the corpus of tweets into 2 subcorpora (original tweets and tweets of other Twitter users retweeted by @CDC_AMD). Regression models were then applied to each subcorpus to compute the probability ratios, to evaluate whether sharing images or videos (or their links) increased the probability of a tweet being retweeted, and whether contents might be a confounder or an effect modifier. Negative binomial regression models were used, as the retweet frequency was overdispersed. Negative binomial regression models took into account data points that were zeros. Hurdle models that take into account the excess of zeros (because many tweets did not have any retweet) were also attempted. A hurdle model is a model with 2 components: (1) a hurdle component for zero versus positive counts and (2) a truncated count component for positive counts [23]. In our hurdle models, the default binomial distribution (logistic regression) was used for the *hurdle component*; a truncated negative binomial distribution was chosen for the *count component*, given the overdispersion of the data.

### Original Tweets by @CDC_AMD

For the original tweet subcorpus, we performed model selection between 5 different statistical models. We started with a Poisson regression model with both the variable for photo or video attachment (*Media*) and the content variable (*Content*) as predictor variables and retweet count as the outcome variable (Model A). Due to overdispersion of the retweet count data, we explored the use of negative binomial regression models (Models B and C) and the hurdle models (Models D and E), and we explored whether content is a confounder by comparing models that include both Media and Content (Models B and D) and those that only include the Media predictor (Models C and E), through likelihood ratio tests and Akaike Information Criterion (AIC).

### Retweets by @CDC_AMD

We constructed 2 negative binomial regression models. Model F included both Media and Content variables as predictors; Model G included Media only.

### Ethics Approval

This study was approved (H15083) by Georgia Southern University’s Institutional Review Board under the B2 exempt category because of the fact that the social media posts analyzed in this study are considered publicly observable behavior.

### Availability of Data and Material

The datasets used and/or analyzed during this study are available from the corresponding author on reasonable request.

## Results

### Findings

As of November 21, 2016, @CDC_AMD had published 768 tweets, of which 88.8% (682/768) were original and 11.2% (86/768) were @CDC_AMD’s retweets of other Twitter users’ tweets. @CDC_AMD had 809 followers, and it followed 195 Twitter accounts. There were 37 likes.

### Followers

Among the 809 followers, 26.0% (210/809) were individual health professionals, 11.6% (94/809) nongovernmental organizations, 3.3% (27/809) government agencies’ accounts, 3.3% (27/809) accounts of media organizations and journalists, and 0.9% (7/809) academic journals. In addition, 54.9% (444/809) of the followers did not fall under any prespecified categories.

### Content

Approximately half of @CDC_AMD’s tweets (360/768, 46.9%) referred to the CDC AMD website and research that was taking place during that time; of these tweets, the majority (354/360, 98.3%) were original tweets (Table 1). Nearly 1 in 5 (135/768, 17.6%) tweets referred to publications of CDC AMD scientists (usually their abstracts on PubMed), of which all, except 2 (133/135, 98.5%), were original tweets. Moreover, 8 in 10 (69/86, 80%) tweets retweeted by @CDC_AMD fell into the miscellaneous category.

Tweets by @CDC_AMD that fell into the miscellaneous category related to a variety of scientific activities that were not included in the prespecified categories. For example, 1 tweet promoted an exhibition in the CDC Museum: “Join us this Thu, Feb 4, 6-8 pm, to see AMD in action in @CDC Museum’s opening of Places & Spaces: Mapping Science. https://t.co/Wp3eZVleUe.” (February 2, 2016; Tweet ID: 694581586566717440); another highlighted the program’s annual science event, AMD Day: “Thanks to @APHL state partners and all who attended #AMDDay2016. ” (September 26, 2016; Tweet ID: 780511838639448064).

Tweets retweeted by @CDC_AMD that fell into the miscellaneous category covered various scientific topics tweeted by other CDC Twitter handles such as @CDCgov, @DrFriedenCDC, and @CDC_NCEZID, as well as tweets posted by other scientific organizations. For example, the most retweeted tweet in our dataset of 768 tweets was about the Google Doodle on scientist Antoine van Leeuwenhoek. On October 24, 2016, @CDC_AMD retweeted a tweet tweeted by the American Society of Microbiology, “RT @ASMicrobiology: Proud to see today’s Google Doodle commemorating the birth of one of the founders of our science Antoine van Leeuwenh...” (The tweet ID of the original tweet by @ASMicrobiology: 790551432734842880; the retweet by @CDC_AMD: 790618487421042688). Regarding photos or videos attached to tweets, 43% (333/768) of the tweets contained photos or videos, whereas the remaining 57% (435/768) did not.

### Retweets

For the subcorpus with original tweets, the results of our model selection process are presented in Table 2. A likelihood ratio test between Model A (Poisson regression) and Model B (negative binomial regression) gave a significant result (χ^2^_1_=259.7, *P*<.001), indicating that Model B, with a higher log-likelihood, fit the data better. A likelihood ratio test between Model B (negative binomial regression with “Content” as confounder) and Model C (negative binomial regression without “Content”) found that there was no significant difference in their log-likelihood (χ^2^_5_=9.3, *P*=.10). The simple model (Model C) had a lower AIC score and was therefore preferred.

Given the excess of zeros in the data, we explored the hurdle models as aforementioned. A likelihood ratio test between Model D (hurdle model with “Content” as confounder; Table 3) and Model E (hurdle model without “Content”; Table 4) found that there was no significant difference in their log-likelihood (χ^2^_10_=17.9, *P*=.06). With a lower AIC score, Model E was therefore preferred (Table 2). Finally, a likelihood ratio test between Model C (negative binomial model) and Model E (hurdle model) found that there was no significant difference in their log-likelihood (χ^2^_2_=0.7, *P*=.70). As Model C has a lower AIC, we chose Model C as the final model for the subcorpus of original Twitter content posted by @CDC_AMD (Table 2).

An original tweet from @CDC_AMD sharing images or videos (or their links) had 37% more retweets (Model C: probability ratio=1.374, 95% CI 1.129-1.674, *P*=.002) than that of an original tweet that did not share images or videos (or their links; Table 3). We observed no significant difference between the content categories, except for the miscellaneous category that has 36% more retweets than tweets referred to the CDC AMD website (Model B: probability ratio=1.355, 95% CI 1.035-1.778, *P*=.03; Model D: probability ratio=1.501, 95% CI 1.046-2.153, *P*=.03).

**Table 1:** Frequency of tweets by their content category.

Content category	Original tweets by @CDC_AMD, n (%)	Retweets of other Twitter users’ tweets by @CDC_AMD, n (%)	All tweets posted by @CDC_AMD, n (%)
Tweets that refer to the CDC^a^ AMD^b^ website	354 (51.9)	6 (7)	360 (46.9)
Tweets that refer to publications of CDC AMD scientists (usually their abstracts on PubMed)	133 (19.5)	2 (2)	135 (17.6)
Training: announcement of webinars, every quarter, collaborated with APHL^c^	26 (3.8)	2 (2)	28 (3.6)
Training: announcement of CDC Bioinformatics fellowship program, collaborated with APHL	29 (4.3)	4 (5)	33 (4.3)
CDC AMD scientists’ activities, such as their visit to a state	15 (2.2)	3 (3)	18 (2.3)
Miscellaneous: anything that does not belong to the aforementioned categories	125 (18.3)	69 (80)	194 (25.3)
Total	682 (100.0)	86 (100)	768 (100.0)

^a^CDC: Centers for Disease Control and Prevention.

^b^AMD: advanced molecular detection.

^c^APHL: Association of Public Health Laboratories.

**Table 2:** Number of parameters, log-likelihood, and Akaike Information Criterion for the 5 models that we tested for the corpus of original tweets created by the @CDC_AMD Twitter profile.

Model	A	B	C	D	E
Model choice	Poisson	Negative binomial	Negative binomial	Hurdle^a^	Hurdle^a^
Predictors^b^	Media+Content	Media+Content	Media only	Media+Content	Media only
Number of parameters	8	9	4	16	6
Log-likelihood (df^c^)	−1210.125 (df=7)	−1080.27 (df=8)	−1084.925 (df=3)	−1075.625 (df=15)	−1084.567 (df=5)
Akaike Information Criterion	2434.25	2176.5	2175.9	2181.25	2179.133

^a^Hurdle model (count=negative binomial; zero hurdle=logistic).

^b^Media: attachment of a photo or a video (or a link to a photo or a video).

^c^df: degrees of freedom.

**Table 3:** Probability ratios for an original @CDC_AMD tweet being retweeted in a negative binomial regression model that includes both the variable for photo or video attachment and the content variable (Model B), and one without content variable (Model C); in a hurdle model that includes both the variable for photo or video attachment and the content variable (Model D) and one without the content variable (Model E).

Explanatory variables of each model			Probability ratio (95% CI)	*P* value
**Model B (negative binomial model)**				
	Contained a photo or video		1.406 (1.114-1.775)	.004
	**Content**			
		Referred to the CDC^a^ AMD^b^ website	Reference	-
		Publication in content	0.874 (0.638-1.197)	.40
		Webinar in content	1.081 (0.643-1.832)	.77
		Bioinformatics in content	1.381 (0.834-2.311)	.21
		Scientist in content	1.066 (0.544-2.120)	.85
		Miscellaneous content	1.355 (1.035-1.778)	.03
**Model C (negative binomial model)**				
	Contained a photo or video		1.374 (1.129-1.674)	.002
**Model D (hurdle model)^c^**				
	Zero or positive (logistic): Contained a photo or video		1.643 (1.137-2.374)	.008
	**Zero or positive (logistic): Content**			
		Referred to the CDC AMD website	Reference	-
		Publication in content	1.179 (0.740-1.878)	.49
		Webinar in content	1.329 (0.579-3.046)	.50
		Bioinformatics in content	2.260 (0.965-5.296)	.06
		Scientist in content	1.685 (0.557-5.097)	.36
		Miscellaneous content	1.150 (0.743-1.782)	.53
	Positive count (negative binomial): Contained a photo or video		1.257 (0.917-1.724)	.16
	**Positive count (negative binomial): Content**			
		Referred to the CDC AMD website	Reference	-
		Publication in content	0.652 (0.417-1.022)	.06
		Webinar in content	0.929 (0.463-1.867)	.84
		Bioinformatics in content	0.999 (0.511-1.950)	>.99
		Scientist in content	0.774 (0.314-1.907)	.58
		Miscellaneous content	1.501 (1.046-2.153)	.03
**Model E (Hurdle model)^c^**				
	Zero or positive (logistic): Contained a photo or video		1.442 (1.059-1.963)	.02
	Positive count (negative binomial): Contained a photo or video		1.344 (1.021-1.770)	.04

^a^CDC: Centers for Disease Control and Prevention.

^b^AMD: advanced molecular detection.

^c^Hurdle models include two model components: a logistic model and a negative binomial model.

Regarding tweets retweeted by @CDC_AMD, we did not observe any significant difference in the retweet count between different contents in Model F (negative binomial regression model with both Media and Content variables). A likelihood ratio test between Model F and Model G (negative binomial regression model with and without Content variables) found no significant difference between the two (χ^2^_5_=8.2, *P*=.14). We could not reject the null hypothesis that content was not a confounder, and we therefore selected Model G that was simpler and had a lower AIC (Table 4). There was no difference in the retweet count between a tweet sharing photos or images (or their links) and one that did not (Model G: probability ratio=0.825, 95% CI 0.508-1.369, *P*=.44; Table 5).

**Table 4:** Number of parameters, log-likelihood, and Akaike Information Criterion for the 2 models that we tested for the corpus of retweets created by the @CDC_AMD Twitter profile.

Model	F	G
Model choice	Negative binomial	Negative binomial
Predictors^a^	Media+Content	Media
Number of parameters	9	4
Log-likelihood	−309.15	−313.27
Akaike Information Criterion	634.2917	632.5408

^a^Media: with a photo or a video (or a link to a photo or a video).

**Table 5:** Probability ratios for a tweet retweeted by @CDC_AMD being retweeted in a negative binomial regression model that includes both the variable for photo or video attachment and the content variable (Model F), and one without the content variable (Model G).

Explanatory variables of each model			Probability ratio (95% CI)	*P* value
**Model F (negative binomial model)**				
	Contained a photo or video		0.703 (0.424-1.184)	.18
	**Content**			
		Referred to the CDC^a^ AMD^b^ website	Reference	-
		Publication in content	3.121 (0.584-25.465)	.21
		Webinar in content	0.882 (0.160-7.304)	.89
		Bioinformatics in content	0.695 (0.170-3.136)	.62
		Scientist in content	1.171 (0.269-6.313)	.84
		Miscellaneous content	2.527 (0.890-6.022)	.053
**Model G (negative binomial model)**				
	Contained a photo or video		0.825 (0.508-1.369)	.44

^a^CDC: Centers for Disease Control and Prevention.

^b^AMD: advanced molecular detection.

## Discussion

### Principal Findings

The AMD program uses Twitter to communicate its accomplishments, provide updates on activities, and share scientific data. This study confirms OAMD’s experience that original tweets containing images or videos (or their links) created for the @CDC_AMD Twitter profile received more retweets. The number of retweets was similar across content topics posted by @CDC_AMD. Our case study of a Twitter handle specializing in communicating public health applications of AMD provides concrete evidence that informs public health communication in practice.

As of November 2016, 54.9% (444/809) of @CDC_AMD’s followers did not belong to any of the prespecified categories. The a priori–defined categories were categories of target audience specified in OAMD’s existing communication strategies. Our results indicated that @CDC_AMD has reached an audience beyond its initial target audience. Anecdotal evidence suggests that these miscellaneous followers could potentially be members of the public who are interested in science and in public health, including university students. Further research will investigate who these people are. By identifying followers who retweeted @CDC_AMD’s tweets, @CDC_AMD may engage them for help disseminating information across Twitter.

Our study found that 51.9% (354/682) of original tweets from @CDC_AMD directed users to the website of OAMD. It suggests that OAMD relied on their website to communicate scientific information in details, whereas they used Twitter to alert users to updates of their website contents. OAMD also promoted their scientists’ research papers; 19.5% (133/682) original @CDC_AMD tweets fell in that category. Our study also found that @CDC_AMD did tweet on topics other than the prespecified content categories, by both tweeting their own tweets and retweeting others’ tweets. It showed scientific and event information not captured in the prespecified content categories defined by OAMD, but that were deemed relevant to their @CDC_AMD followers by OAMD at an ad hoc basis. Future research into the tweets in the miscellaneous category would help OAMD detail these tweets and develop new content categories for the playbook of their routine communication strategies.

### In the Context of the Literature

Previous research has identified factors that contributed to more retweets. Suh et al found that the presence of hashtags and URLs in a tweet and the number of followers and friends (users whom one follows) of a Twitter user were positively associated with the probability of a tweet being retweeted [24]. Can et al found that the ratio between followers and friends was highly correlated with retweet count [25]. Soboleva et al noted that hashtags and retweet requests were associated with higher retweet rates, whereas URL links and mentions were associated with lower rates of retweeting [26].

In this study, we focused on the effect of attaching images or videos to tweets on their retweet frequency, in particular, tweets tweeted by @CDC_AMD. According to Soboleva and colleagues [26], images can increase the effect of advertisements partly because they can convey meanings not expressed via words. In their literature review, Soboleva et al [26] also highlighted that images can influence consumer persuasion, have the potential to effect attitude change, and increase recall of advertisements’ verbal information. Prior studies on the effect of links to visual cues on retweet frequency using Twitter data collected in 2011 had conflicting results [27,28]. Analyzing tweets associated with tourism in European cities, Bruni et al [27] found that tweets with a link to a photo or a video had more retweets than those without, and tweets linking to a photo had more retweets than tweets linking to a video. On the contrary, Malhotra et al [28] could not identify statistically significant effect of embedded links to websites, photos, or videos on retweet likelihood of tweets tweeted by 47 major commercial brands. However, another study that analyzed tweets from 2009 to 2012, drawn from 298 Twitter profiles operated by 100 top brands, found that links to photos or videos increased the likelihood of a tweet being retweeted [29]. A more recent study analyzing 2014 Twitter data posted by 32 major commercial brands found that photos were consistently associated with higher retweet rates across 3 industries under study (automobile, fast-moving consumer goods, and luxury) [26]. Nevertheless, the same study found that the industry of the brand was an effect modifier for the effect of videos on retweet rates (significant increase in retweet probability for luxury brands, but insignificant effect for the other 2 industries) [26]. Similar research on Sina Weibo (a Chinese social media platform similar to Twitter) also found that multimedia Weibo posts received more reposts, and were reposted by users for a longer period of time, than text-only Weibo posts [30]. In a retrospective observational study, the effect of attaching visual cues to a tweet on its retweet frequency was found to vary across cycles of original tweets with hashtags #CDCGrandRounds and #VitalSigns. The probability ratios of retweet frequency of tweets with visual cues as compared to tweets without visual cues ranged from less than 1 to as high as 34, depending on the topic of the specific CDC Grand Round event or Vital Signs publication [31]. In a prospective, case-control crossover study of visual abstracts (graphics that summarize the main message of a scientific paper), a surgical journal found that the retweet frequency of its tweet that carries both the title of the paper and a visual abstract was 8.4 times that of a tweet with the title of the paper only (92 vs 11 retweets, *P*<.001) [32]. Further evidence was provided by Can et al who identified certain features of an image that were positively correlated with retweet count [25]. Consistent with prior findings, this study on @CDC_AMD adds evidence to a growing literature that attaching visual cues to tweets will increase their retweet frequency, and this practice may enhance Twitter users’ engagement with health communication messages promoted by medical and public health professionals.

### Limitations

Our study is limited to its cross-sectional design and to 1 CDC Twitter profile. The strength of this study is that we manually coded and statistically analyzed the entire corpus of tweets published by @CDC_AMD. Although our study follows the protocol defined a priori, it lacks the ability to classify 55% of the followers and 80% of contents retweeted by @CDC_AMD that did not fall into any of the prespecified categories. Further research on the followers is warranted. We select retweet frequency as our measure of engagement of a tweet, while acknowledging its limitations. We acknowledge that other measures of impact exist, including “reach,” defined as the sum of the potential number of individuals exposed to each retweet of a tweet. While “reach” may account for the number of followers who retweeted a tweet, retweeting is a high level of engagement, and retweet frequency provides solid evidence to our research questions. We did not distinguish videos from photos when we manually coded the visual cues. Future research can further investigate the difference in effect between these 2 types of visual cues for a public health Twitter handle, as compared with results for Twitter handles of major commercial brands [26]. We also did not analyze specific visual features of the photos or videos (as in [25]) that were beyond the scope of our study. There were other potential confounders or effect modifiers [26] that were not included in our study. Given the nature of this study as an observational study, we cannot rule out the possibility of residual confounding by other factors.

### Conclusions

As part of its communication strategies, OAMD includes its Twitter handle, @CDC_AMD, as one of its communication channels to its audience. This study underscored the importance of including visual information to build engagements with @CDC_AMD tweets. Following on this brief study conducted in mid-November of 2016, OAMD increased its use of visual cues for tweets, including photos and graphs, and we have seen steady engagement rates during the subsequent 12 months.

On the basis of anecdotal evidence that Twitter is used highly in the biotechnology and biomedical industries, OAMD decided to use this mechanism to target these audiences. On the basis of monthly reviews of Twitter Analytics, audience areas of interest demonstrate that @CDC_AMD hits the intended audiences (eg, 75% are interested in science news, 70% in tech news, and 70% in biotech and biomedical). Further investigation is needed to identify the 55% of @CDC_AMD’s followers who did not belong to any of the prespecified categories in this study. These 444 followers could include personal Twitter handles of followers within the target audience and used outside their professional realm. However, a more in-depth study or survey is necessary to determine their interest in @CDC_AMD.

## Abbreviations

AIC: Akaike Information Criterion
AMD: advanced molecular detection
APHL: Association of Public Health Laboratories
CDC: Centers for Disease Control and Prevention
DPEI: Division of Preparedness and Emerging Infections
HEMU: Health Economics and Modeling Unit
NCEZID: National Center for Emerging and Zoonotic Infectious Diseases
OAMD: Office of Advanced Molecular Detection
RQ: research question
UTC: Universal Coordinated Time
